# Composition Influences the Pathway but not the Outcome of the Metabolic Response of Bacterioplankton to Resource Shifts

**DOI:** 10.1371/journal.pone.0025266

**Published:** 2011-09-27

**Authors:** Jérôme Comte, Paul A. del Giorgio

**Affiliations:** Groupe de Recherche Interuniversitaire en Limnologie (GRIL), Département des sciences biologiques, Université du Québec à Montréal, Montréal, Québec, Canada; Universidad Miguel Hernandez, Spain

## Abstract

Bacterioplankton community metabolism is central to the functioning of aquatic ecosystems, and strongly reactive to changes in the environment, yet the processes underlying this response remain unclear. Here we explore the role that community composition plays in shaping the bacterial metabolic response to resource gradients that occur along aquatic ecotones in a complex watershed in Québec. Our results show that the response is mediated by complex shifts in community structure, and structural equation analysis confirmed two main pathways, one involving adjustments in the level of activity of existing phylotypes, and the other the replacement of the dominant phylotypes. These contrasting response pathways were not determined by the type or the intensity of the gradients involved, as we had hypothesized, but rather it would appear that some compositional configurations may be intrinsically more plastic than others. Our results suggest that community composition determines this overall level of community plasticity, but that composition itself may be driven by factors independent of the environmental gradients themselves, such that the response of bacterial communities to a given type of gradient may alternate between the adjustment and replacement pathways. We conclude that community composition influences the pathways of response in these bacterial communities, but not the metabolic outcome itself, which is driven by the environment, and which can be attained through multiple alternative configurations.

## Introduction

After decades of research on microbial processes in aquatic systems there is now evidence that bacterioplankton communities are extremely sensitive and reactive to changes in environmental conditions [1]. For example, even slight changes in resources (nutrients, organic matter) and conditions (e.g. salinity, temperature) often elicit large responses in terms of community metabolism in both marine [2] and freshwater [3] bacterial communities. The direction and magnitude of change in this overall metabolic response have been intensively studied and is in general relatively well understood [4]. Less well understood are the mechanisms involved in this response. One interesting feature of bacterioplankton communities is that total cell abundance (and biomass) tends to vary much less, both spatially and temporally, than either bacterioplankton metabolism, or the environmental factors that influence bacteria. For example, bacterial abundance in temperate lakes generally ranges from 1 to 6×10^6^ cells ml^−1^, and yet community growth rates and bacterial production may vary by several orders of magnitude [5]. The same pattern has been observed in marine systems [6]. If the change in community metabolism is not primarily driven by shifts in abundance or biomass, it follows that there must necessarily be profound changes in other aspects of community structure [6]–[8].

The structure of bacterioplankton communities is complex and includes a wide range of properties, such as the morphological and metabolic characteristics of individual cells, and the relative distribution of these within the community. Changes in overall community metabolism may result from shifts in the total number or size of cells, in the intrinsic level of activity of the cells, in the proportion of cells with different levels of activity, or most likely, by a combination of the above [6]. In turn, these shifts in cell abundance, morphometry and physiological state may or not be associated to changes in the composition of the community. Determining the role that community composition plays in shaping the response of bacterioplankton to environmental change is indeed one of the central questions in contemporary microbial ecology [9]–[10], but this role has typically been elusive to discern, precisely due to the difficulty in untangling the concomitant shifts that occur at other levels of community structure. This is the objective of the work presented here.

### Conceptual approach

**Figure 1:**
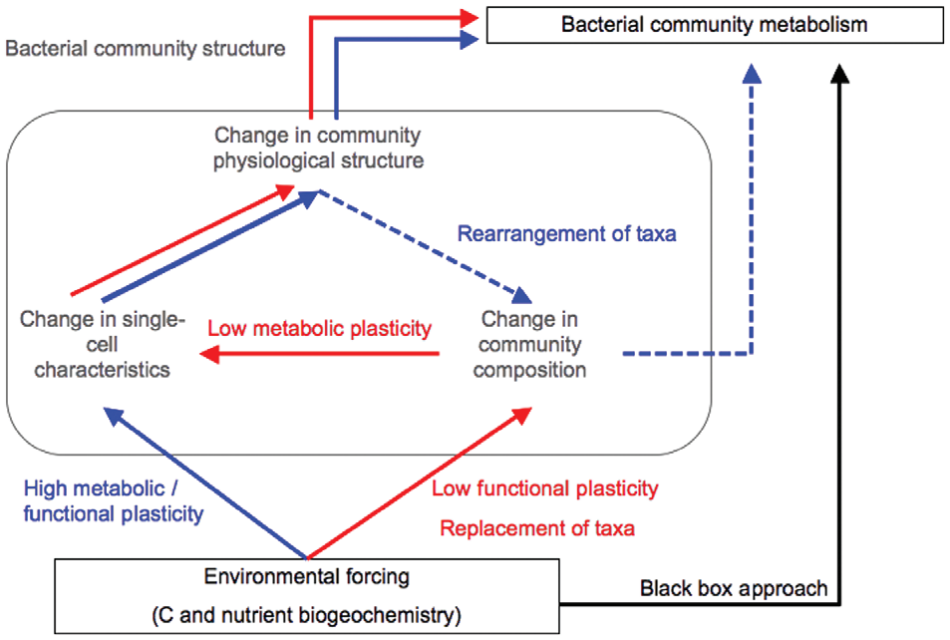
Conceptual scheme of the components of the response of freshwater bacterial communities to changes in resources. In contrast to the classical black box approach (black line), this scheme assumes two major pathways in this response, both of which mediated by shifts in different aspects of community structure: In the Adjustment pathway (full lines in blue), changes in resources trigger shifts in the level of activity and other cellular properties of the existing dominant phylotypes. In this scenario, change in community composition involves shifts in the relative abundance of the existing taxa, but not their identity (i.e. rearrangement, dashed blue line). In the Replacement scenario (red lines), resource gradients trigger shifts in the composition of the community, in terms of the identity of the dominant phylotypes, which have intrinsically different single-cell characteristics. In both scenarios, the final metabolic response is mediated by shifts in the physiological structure, that is, the distribution of activity and physiological states within the community. In either scenario, the response may or not include changes in biomass or cell abundance. The Adjustment scenario is associated to either modest environmental changes, or to communities that are more collectively more plastic. The Replacement scenario is associated to either steep environmental gradients, or to communities that are collectively less plastic. In this context of this scheme, functional plasticity refers to the breadth of capacities to perform specific processes, for example, the forms of N or the specific organic substrates that can be used at the community level. Metabolic plasticity, on the other hand, refers to the breadth of metabolic activity, in terms of rates of major processes such as respiration and growth, for example, associated to any given bacterial community composition, under varying environmental conditions. We quantitatively tested variations of these two basic scenarios using path analysis (see Figure S1).

Schimel and Gulledge [11] presented a conceptual framework to investigate how global change may alter soil microbial processes. They proposed two main scenarios: Environmental change can either alter the functioning of the existing organisms, or alter the structure of the community by modifying the existing taxa, thereby changing key physiological characteristics that drive community performance. More recently, Allison and Martiny [10] extended this conceptual framework to aquatic microbial communities in general, by incorporating the notions of functional redundancy, resilience and resistance at the community level. We build on these frameworks, and in this study we have conceptualized the response of freshwater bacterial communities to changes in the environment into two simplified scenarios: The response can be initiated by changes (1) at the single-cell level, in terms of metabolic adjustments of the existing phylotypes (referred to as Adjustment scenario), or (2) at the compositional level (referred to as Replacement scenario) (Figure 1). The adjustment scenario corresponds to situations wherein the dominant phylotypes are generalists with a high degree of plasticity, rendering the community more “resistant”, sensu Allison and Martiny [10], and/or the degree of environmental change is small relative to the existing metabolic breadth in the community. The resulting adjustments at the level of single-cell activity then propagate to determine shifts in the physiological structure of the community, in terms of the distribution of cells with different levels of activity, which in turn determine the overall metabolic performance of the community (Figure 1).

In the Replacement scenario, the dominant taxa cannot accommodate the changes in the environment due to either a lower overall level of plasticity, rendering the community less “resistant”, sensu Allison and Martiny [10], and/or to stronger environmental gradients. This response in community composition occurs as changes in the actual identity of the dominant phylotypes, conceivably through selective activation and inactivation of phylotypes already present in the metacommunity pool. In this replacement scenario, changes in the identity of taxa generate shifts in the single-cell characteristics, which would then induce shifts in the physiological structure and eventually changes in terms of community metabolism.

The conceptual model shown in Figure 1 no doubt represents an oversimplification, since both scenarios probably coexist, but it does provide a framework to explore the relative importance of compositional changes versus metabolic versatility in shaping the response of bacterial communities to environmental forcing, and how this may change both across types and intensities of gradients, and temporally within a given system. In order to quantitatively assess the pathways shown in Figure 1, we must be able to establish connections between the various components of community structure, and between these and both the environment and community metabolism. In a previous paper [12] we showed that the connections between the absolute patterns in the various components of structure, including community composition and functional capacities, were often weak or non-existent. This does not mean that these components are uncoupled, and in later studies we have shown that community composition and functional capacities are in fact highly correlated to each other, but only in terms of their rates of change along environmental gradients, not in their absolute patterns [13].

In this paper, we build on the approach and results described in Comte and del Giorgio [13] to examine how the rates of change in four key components of community structure relate to each other, to shifts in the main resources and to changes in community metabolism, and in turn we use these relationships to quantitatively explore the two main scenarios proposed in Figure 1, using structural equation modeling. We further assess whether these pathways change with the intensity of environmental gradients and with time. We selected a temperate watershed that contains a diversity of freshwater habitats (lakes, rivers and marshes) that are interconnected, and focused within this watershed on a series of environmental transitions (i.e. the interfaces between the major aquatic habitat types) that differ in the nature and intensity of the resource gradients involved, thus generating a range of potential outcomes in terms of bacterial successions originating from the same regional metacommunity [13].

We targeted four major components of bacterioplankton community structure: Bacterial abundance (BA), community composition (BCC), the distribution of physiological states (PS), and single-cell characteristics (SCC) of bacteria (Table 1). Similarly, the end-point response was an ensemble of ecologically-relevant aspects of community metabolism (BCM, Table 1). In turn, the environmental gradients were described by a set of environmental variables (RES, Table 1). We thus generated three matrices (BCC, PS and SSC) plus an individual variable (BA) that describe community structure; one matrix that describes the overall metabolic response (BCM), and a resource matrix (RES) that characterizes the actual environmental transitions. We determined the rates of change in the six categories along each of these transitions, and then used the resulting relations between these rates of change to reconstruct the sequence of events using structural equation modeling.

**Table 1:** Definition of terms used in the article and list of variables measured to describe them.

Terms	Definitions	Variables
**Bacterial community metabolism (BCM)**	Bulk community metabolic rates and metabolic performance	Rates of bacterial biomass production (BP, ^3^H-Leucine), growth (BGR, ^3^H-Thymidine), ratio BP∶BGR, respiration, growth efficiency, carbon demand, ATP content
**Bacterial physiological structure (PS)**	Distribution of metabolic activity and cell-specific physiological and morphometric characteristics within the community	Specific rates of BP, BGR, respiration, ATP content; proportion of high-DNA, respiring, injured, intact and dead cells
**Bacterial single-cell characteristics (SCC)**	Average cell properties within the community, including size, DNA content and cytometric characteristics	Fluorescence and side scatter values of HNA, respiring cells, fluorescence values of injured, intact and dead cells
**Bacterial community composition (BCC)**	The community 16S-rDNA fingerprint based on DGGE	The DGGE banding pattern and the relative contribution of each band to the total intensity of the lane

## Results

### Metabolic response of bacterial communities to environmental gradients

**Figure 2:**
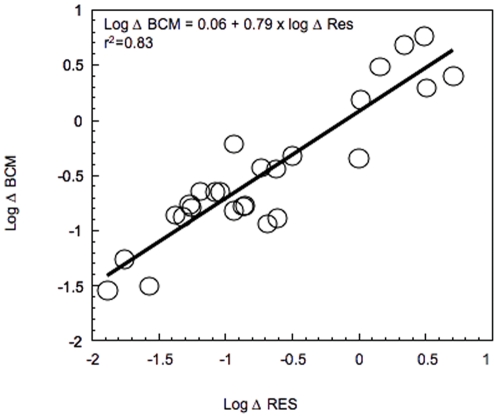
Rates of change in bacterial community metabolism (Δ BCM) as a function of the rate of change in resources (Δ RES) along the different aquatic transitions sampled. The rates of change were calculated relative to the water transit time within each transition. Each point represents the rate of change determined for an individual transition and for a single date from June to August 2005. The data are log10-transformed, and the line represents the least square regression fit.

The 13 transitions studied differed greatly in the type and intensity of the environmental gradients involved, and also in the temporal and spatial scales at which they occur (Table S1). Along the different transitions, the rate of change in the ensemble of metabolic variables was strongly positively correlated to the rate of change in the ensemble of resources, suggesting that the steeper the resource gradient along a transect, the greater the changes in BCM along the same transect (Figure 2).

### Response of community structure to changes in resources

**Figure 3:**
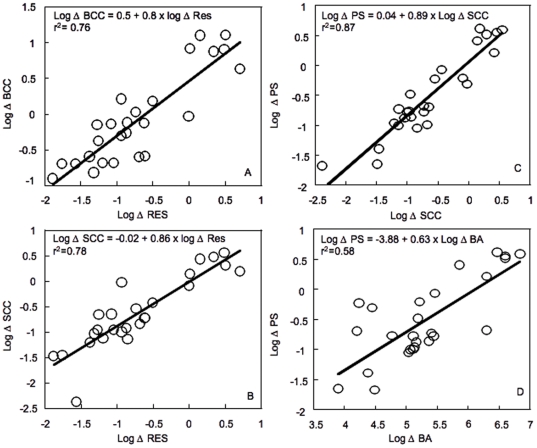
Rates of change in bacterial community composition (Δ BCC, Figure 3A) and single-cell characteristics (Δ SCC, Figure 3B) as functions of rates of change in resources (Δ RES); rates of change in bacterial community physiological structure (Δ PS) as a function of rates of change in bacterial single-cell characteristics (Δ SCC, Figure 3C) and abundance (Δ BA, Figure 3D). Each point represents the rates of change determined for an individual transition and for a single date from June to August 2005. The data are log10-transformed, and the line represents the least square regression fit.

The four components of community structure also appeared to respond to changes in resources along these transitions, and the rates of change for BA, PS, SCC and BCC were positively correlated to the rate of change in resources, with the highest rates of change in both composition and single-cell characteristics (Figures 3A and 3B). The magnitude of change of these various aspects of community structure varied seasonally as well for any given site, and whereas the rate of change in SCC and BA varied by around 3 orders of magnitude, the rates of change in BCC varied only by 2 orders of magnitude along the same gradients over the entire study period (Figures 3A and 3B).

### Relationships between components of bacterial community structure

The rates of change of the four components of community structure were significantly correlated to each other, albeit with very different degrees of strength. For example, changes in PS were strongly correlated to changes in SCC (Figure 3C), but much less so to changes in BA (Figure 3D), such that the highest rates of change in PS were mostly related to steep shifts in SCC than in total abundance. Likewise, the rates of change in BCC were positively correlated to both SCC and PS, but the relationship was much stronger with the former suggesting that changes in BCC results in changes in the intrinsic properties of cells (data not shown).

### Relationship between in the components of community structure and the overall metabolic performance of the community

**Figure 4:**
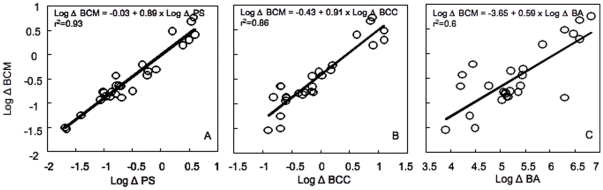
Rates of change in bacterial community metabolism (Δ BCM) as a function of the rates of change in bacterial community composition (Δ BCC, Figure 4A), physiological structure (Δ PS, Figure 4B), and bacterial abundance (Δ BA, Figure 4C) along the same transitions. Each circle represents the rates of change determined for an individual transition and for a single date from June to August 2005. The data are log10-transformed, and the line represents the least square regression fit.

The rates of change in BCM were more strongly correlated to the rates of change in PS (Figure 4A) and to BCC (Figure 4B), whereas the relationship with the rates of change in BA was much weaker (Figure 4C) suggesting that changes in the overall community performance were more related to changes in the physiological structure and composition of the community than in total abundance. Interestingly, at low rates of change in BCC, the link with changes in BCM appears to weaken.

### SEM analysis of pathways of community response to environmental change

**Figure 5:**
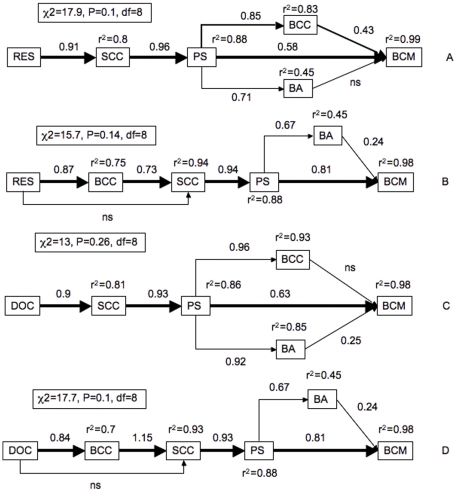
Results of structural equation modeling to explore potential pathways leading from changes in resources (Δ RES) to the final bacterial metabolic response (Δ BCM). Figures 5A and B show the pathways that were significant for the various months of study, based on the changes in the overall resource matrix: The model that was significant for July (B-DAG4, Figure 5A) followed the Adjustment scenario (response mediated by shifts in single-cell activity and characteristics), whereas the model that was significant for August (A-DAG4, Figure 5B) followed the Replacement scenario (response mediated by shifts in community composition). Figures 5C and D show the pathways that were significant based on changes in dissolved organic carbon (Δ DOC) alone, rather than on the ensemble of resources: In June there was a significant model (B-DAG4) that followed the Adjustment scenario (Figure 5C), whereas in August there was a significant model (A-DAG4) that followed the Replacement scenario (Figure 5D). Arrows represent causal relationships between variables with the value of the standardized path coefficient (the standard deviation change of a variable given a standard deviation change the corresponding causal variable). Arrows in bold represent the most probable sequence between the different variables. Coefficients refer to the significance of the F statistic (i.e. different from 0) showing that the causal relationship cannot be rejected. NS represents non-significant causal relationships. The proportion of variation explained for each variable (r^2^) takes into account the total effect (direct and indirect) of the ensemble of the preceding variables.

Our SEM analyses based on the entire data set resulted in the rejection of all nine alternative models that we tested. We explored the possibility that the pathways may differ temporally by testing the nine alternative models for each of the three sampling periods separately, and this analysis resulted in very different patterns for the three months: All nine models were rejected for the month of June, although the individual variables were highly correlated to each other in this period. In July, all the models related to Scenario A were rejected, but one of the models from Scenario B (B-DAG4, see Figure S1) was significant (χ^2^ = 17.9, p = 0.1, df = 8), (Figure 5A). The variability in the different factors was well explained by the structure of B-DAG4 (78 to 97% explained), except for BA (51%). The path coefficient between BA and BCM was not significant suggesting that changes in BA did not have a significant direct effect on changes in BCM (Figure 5A). Interestingly, in this pathway BCC is not within the main sequence leading from resources to BCM, but changes in BCC were nevertheless significantly related to both changes in PS and BCM (Figure 5A). Finally, for August all the models related to Scenario B were rejected, but one of the models for Scenario A (A-DAG4) was significant (χ^2^ = 15.7, p = 0.14, df = 8, Figure 5B). Changes in BA explained only a very small fraction of the variations in BCM, and as was observed in July, and the proportion of the variance in BA explained by the model was low (45%).

We further tested whether the type of predominating resource gradient might influence the pathways of response, by considering the changes in the individual resources rather than in the overall resource matrix. Interestingly, the results now show that in June, one of the structures associated to Scenario B (B-DAG4) was highly significant (χ^2^ = 13, p = 0.26, df = 8) based on changes in DOC alone (Figure 5C), and all variables were well described by the model (from 81 to 98% of variations explained). In July, no DAG based on the changes in individual resources fit the data, suggesting that no single resource variable drove the bacterial response. In August, the same structure that was significant based on the ensemble of resource variables (A-DAG4), was also significant based on changes in DOC alone (χ^2^ = 17.7, p = 0.1, df = 8) (Figure 5D).

Finally, we explored whether the intensity of the resource gradients may influence the pathways of response, by grouping our environmental transitions into two equal-sized categories based on their magnitude of change in resources (high and low), and testing the nine alternative models on each of these groupings separately. All 9 models were rejected for the data in the “Low” environmental change category, but three models, all belonging to Scenario B (B-DAG2, 3 and 4), were significant for the “High” environmental change category, with model B-DAG4 providing the best fit to the data (χ^2^ = 12.4, p = 0.22, df = 8).

## Discussion

One of the main questions in contemporary ecology has been the extent to which the composition and diversity of communities play a role in shaping their overall performance and their responses to environmental forcing [14]–[16]. Microbial ecology studies to date have addressed this issue using either experimental manipulations of communities or resources [9], [17]–[21], or along natural gradients [8], [13], [22]–[23], and have generally focused on very specific links, for example between community composition and environmental parameters [24], growth rate [25], or single-cell characteristics [26].

There are two fundamental assumptions underlying most of these studies: 1) That the link between community composition or diversity and community function or metabolism, if it exists, should be deterministic, such that a particular configuration of the community should correspond to a particular level of metabolic activity or function; and 2) that this potential influence of community composition is exerted directly, such that it can be detected by simple statistical comparisons of composition and metabolism or function. There are strong reasons to think that these two key assumptions are unjustified: On the one hand, natural aquatic bacteria may be extremely plastic [27]–[29], aquatic bacterial communities are extremely diverse [30]–[32], and there is evidence for widespread functional redundancy in these communities [33]–[36]; it would be thus extremely unlikely that a particular level of metabolic activity or functional capacity would correspond to one, and only one, community configuration in terms of composition. On the other hand, if community composition were to play a role in the overall metabolic response of the community, this must necessarily be mediated by shifts at the level of the individual cell activity and in the distribution of activity within the community, such that one cannot be understood without considering the other [37].

In this study, we attempted to circumvent these two key assumptions: We do not assume an a priori deterministic relationship between BCC and metabolism, and we do not assume that the potential link between BCC and metabolism is direct, but rather that it is mediated by changes at the single-cell level and at the level of the physiological structure of the community. We confirmed that bacterial community metabolism closely tracks environmental change in these freshwater transitions, which is in agreement with previous reports by us [12] and others [1], [4]. We established that the overall metabolic response of freshwater bacterial communities was not explained by shifts in bacterial abundance, but rather mediated by changes in the community structure. We further established that the four major aspects of bacterioplankton community structure (bacterial abundance, single-cell characteristics, physiological structure and community composition) tended to covary with each other, but only in terms of their rates of change along environmental transitions, not in their absolute patterns. This is important, because had we focused only on the absolute patterns in the various components, rather than on their rates of change, we would probably have not detected links between them, and we would have thus been unable to explore the potential pathways of response.

The two basic scenarios (Adjustment and Replacement) that we tested admittedly represent extremes in the potential response pathways, and there is little question that both likely coexist, and thus the SEM results need to be interpreted with caution: The fact that no structure is significant could suggest that the models selected simply do not reflect the structure of the data, that several pathways coexist at a given time, or that the pathways change in time and space. Our results would suggest the latter. Our path analyses showed that no model fit the entire data set, suggesting that there is no single type of pathway of bacterial response that is prevalent in these aquatic bacterial communities. We found evidence, however, that both the Adjustment and Replacement scenarios actually operate, and in fact, may alternate temporally within a given set of systems. In June there was no model that fit the data, in July an Adjustment model fit the data and the sequence of responses to changes in resources was clearly mediated by shifts at the single-cell level of existing dominant phylotypes. In August the response of the community to shifts in resources appears to have been primarily mediated by the replacement of phylotypes. This model further suggests that the changes in the identity of bacteria led to changes in single-cell characteristics, which then propagated to influence the physiological structure and finally BCM. In support of this conclusion, we observed that the rates of change in the DGGE banding patterns were indeed highest in August, relative to July and June (Figure S2B).

The alternating scenarios that we observed may be the result of changes in the dominant resource to which bacteria are responding. The fact that for June the only significant model was one based on changes in DOC rather than in the ensemble of resources would suggest that at certain times there may be a single, key resource that drives the response of bacterial communities, whereas during other periods bacteria respond to multiple factors. Previous studies have also suggested a major role of DOC in controlling bacterial community performance (e.g. [18], [23], [25], [38]–[39]).

In June, the response of the community to shifts in DOC was driven by adjustments at the single-cell level. The analyses of the rates of change in H′ and of the DGGE banding patterns further suggests that during this period, a rearrangement of the dominant phylotypes, in terms of relative abundance, rather than their replacement predominated (Figure S2). Interestingly, DOC also appeared to play a key role in driving the changes in bacterial metabolism in August, as it did in June, yet the pathway of response was different and followed the Replacement scenario. This would suggest that a similar type of environmental forcing may elicit very different pathways of response at different times, some involving community composition, and some not.

This result may be linked not to the type of gradient per se but rather to its intensity, and we had originally hypothesized that the Replacement scenario should occur under steeper resource gradients, whereas the Adjustment scenario should prevail along less pronounced gradients, but our data do not support this hypothesis. When we separately analyzed transitions with stronger and weaker gradients, we found exactly the opposite result: The data from steeper gradients clearly fit pathways associated to the Adjustment scenario, whereas the weaker gradients did not fit any particular pathway of response. The fact that these bacterial communities appear to be able to respond through metabolic adjustments to even the strongest gradients would suggest that there is a potentially very large degree of metabolic versatility in these communities, most likely as the result of dominance by functional generalists, as has been hypothesized for bacterioplankton communities in general [29], [40]. The question is then, why does the response at times involve changes in BCC, even in circumstance of relatively modest resource change?

We postulate here that the key determinant for the type of response may be linked to intrinsic properties of the communities, specifically related to the level of metabolic and functional plasticity of the dominant phylotypes (see definition of terms in Figure 1). Phenotypic (metabolic and functional) plasticity, defined as the capacity for single genotypes to change their chemistry, physiology, development, morphology, or behavior in response to environmental cues [41], [42], is clearly a property of individual taxa or phylogenetic entities. It has been hypothesized, however, that the degree of plasticity of interacting members of the same community or of interacting players in a food web might not be independent from each other, and that there may be common and reciprocal regulation of phenotypic plasticity within communities and food webs [42].

There are many factors, in addition to resources, that can influence the seasonal phylogenetic succession of bacterioplankton, including temperature or physical conditions [43], and biological interactions, such as grazing [44]–[45] or viral infection [46]–[47]; it is possible that these factors indirectly influence the response of bacterial communities to changes in resources by somehow favoring, at different points of the succession, phylotypes that have different niche breadths and degrees of metabolic plasticity towards the same set of environmental factors (e.g. DOC type and concentration). In this regard, our results could suggest that niche breadth and phenotypic plasticity, as they relate to resource utilization, may be co-selected in the dominant phylotypes, such that some community assemblages may be characterized collectively by an overall higher degree of resistance. Importantly, our results would further suggest that the resource gradients themselves may not be responsible for determining either community composition or its associated level of resistance, such that it is difficult to predict the pathway of response on the basis of the type or intensity of the gradient.

In this conceptual framework, community composition always plays a role in the response, because it is what determines the overall level of community resistance, which in turn determines whether the response to an environmental gradient will be predominantly via the Adjustment or Replacement scenario. A complicating factor in this scheme is that there also appears to be significant functional redundancy in these microbial communities, since we observed a large degree of compositional change that was not linked to either shifts in resources, or to changes in either single-cell characteristics or in physiological structure. This would suggest that there are many possible community configurations that might share a similar level of community resistance.

The framework that we propose has conceptual implications on how we view the links between composition and the functioning of these microbial communities, since it suggests that there may be emergent properties of these communities, such as the community resistance, which are linked to composition but that are regulated by factors that are different that those that influence the overall metabolic performance of the community. It has also practical implications on how we design experiments and field studies to explore potential links between diversity and function in these communities, and especially how we interpret experimental results and empirical patterns observed in natural aquatic systems, because the lack of apparent coupling between composition and function that has often been observed in microbial communities in no way implies that composition does not play a role, but rather that this role probably does not conform to the preconceived notions and assumptions of most of these studies.

## Materials and Methods

### Sampling sites and procedure

Thirteen interfaces within a watershed containing interconnected lakes, rivers and marshes were sampled during the growing season in 2005. Details of sampling locations are provided in Comte and del Giorgio [13]. For each of the 13 environmental transitions (ET), the distance between sampling sites as well as the average transit time (TT) of water masses between two successive sampling points was estimated, as described in Comte and del Giorgio [13].

No specific permissions were required for the locations sampled, except for lake Fraser located in the national park of Mont-Orford and for which the Société des établissements de plein air du Québec (SEPAQ) gave their permission. Owners of lands allowed using their private access to some lakes and marshes. Our field activities did not required any specific permissions and did not involve endangered or protected species.

### Chemical analyses

DOC concentrations were measured with a TIC TOC 1010 Analyzer (OI analytical), DOC absorbance was determined at 280 and 440 nm using a Biochrom spectrophotometer; total phosphorus and nitrogen were measured spectrophotometrically following persulfate digestion.

### Bacterial community metabolism

Bacterial production (BP) was assessed as the rate of incorporation of both ^3^H-leucine, and ^3^H-thymidine into DNA; Bacterial respiration (BR) was determined using membrane-inlet mass spectrometry following procedures provided in Comte and del Giorgio [12]. Estimates of bacterial growth efficiency and bacterial carbon demand (BCD) were derived from measurements of BP and BR. Intracellular ATP concentration was assessed by bioluminescence.

### Bacterial community composition

Bacterial DNA was extracted using CTAB buffer and chloroform/isoamyl alcohol and amplified in PCRs reactions using GC clamp-358 F and 907 rM primers (HPLC purified; Sigma Genosys) [12]. The resulting products were separated into bands by DGGE and further comparison of banding profiles for different samples identified matching bands. Bacterial community diversity was examined by the Shannon index of diversity H′ [48], which was calculated using the number of bands and their relative fluorescence in the DGGE gels. We further assessed the changes in presence–absence of bands at the different locations of each environmental transition in comparison to the banding pattern of the corresponding head location of these transitions.

### Flow cytometry analyses

Details of cytometric approaches are provided in Comte and del Giorgio [12]. Bacterial abundance was assessed using SYTO 13, and High- and Low-DNA cells were discriminated; cells with depolarized and damaged membranes were enumerated using DiBAC4(3) and BacLight Live/Dead viability kit respectively; respiring and polarized cells were enumerated using CTC and DiOC6(3), respectively.

### Construction of dissimilarity matrices

For 3 of the 4 components of community structure (BCC, PS and SCC), in addition to bacterial community metabolism and resources, we constructed a raw data matrix where rows represent sites at each sampling date, and columns correspond to the variables measured for that particular category. For each raw matrix, data were successively normalized and standardized, and then a site dissimilarity matrix was generated based on Euclidean distances, for each of the categories listed in Table 1.

### Calculation of rates of change

This approach has been developed and described in detail in Comte and del Giorgio [13]. Briefly, for each environmental transition (ET), we plotted the dissimilarity between the first sampling point and the successive sampling sites within that transition as a function of the transit time between each sampling point, and used the slope of the resulting least square regression model as an estimate of rates of change (per hour) in the 5 categories (BCM, BCC, SCC and PS). In the case of BA, we estimated the rates of change by plotting the actual difference in abundance along the transition as a function of transition time. We further estimated rates of change in bacterial diversity, on the basis of the difference in estimates of Shannon index of diversity, and rates of change in DGGE banding patterns, by calculating the difference in shared bands between two samples. These latter two categories were not included in path analyses.

### Structural equation modeling

We carried out structural equation modeling (SEM) [49] to identify the sequence of causal relationships that may exist between rates of change in resources, in the components of community structure, and in community metabolism, and in particular, determine the position that composition has in this sequence. In our conceptual framework, changes in resources generate a series of changes that propagate among components of community structure and eventually result in changes in community metabolism; we thus considered changes in resources as the independent, driving variable in all the causal structures (referred to as Directed Acyclic Graphs, DAG), and BCM as the ultimate, dependent response variable. We built structures such that resources and BCM did not share a direct link, and the community structure variables were placed between these endpoints; the models tested varied in terms of the position of these community structure variables relative to the endpoints and to each other. We tested two broad categories of models, i.e. the replacement and adjustment scenario that target the two main pathways described in Figure 1, and which differ mainly in the position of BCC. Within each of these two broad scenarios there are multiple alternative structures, and we tested a total of 9 potential models (see Figure S1 for details). The principle and output interpretation of SEM analyses are explained in Text S1.

## Supporting Information

Figure S1 **Modeling of bacterial response to changes in resources (Δ RES).** Δ BCC, Δ SCC, Δ PS, Δ BA and Δ BCM represent the rates of change in community composition, single-cell characteristics, physiological structure, total abundance and community metabolism respectively. Arrows represent causal links between variables. The same models have been tested with changes in DOC concentration as independent variable. In Scenario A (Replacement scenario), Δ RES induce activation of particular ecotypes that are characterized by single-cell and physiological properties, which directly determine Δ BCM (A-DAG1). Alternative were considered: (i) a direct link between Δ RES and Δ SCC (A-DAG2); (ii) a direct link between Δ PS and Δ BCM (A-DAG3); (iii) a direct link between Δ BCC and both Δ BA and Δ SCC, both of which linked to Δ PS, which in turn determine Δ BCM (A-DAG4). In Scenario B (Adjustment scenario), Δ RES trigger changes in cell activity of the existing phylotypes, which influence the physiological structure (B-DAG1). Alternative were considered: (i) a direct link between Δ BCC and Δ BCM (B-DAG2), (ii) Δ BA mediates the link between Δ PS and Δ BCM independently from Δ BCC (B-DAG3), (iii) a direct link between Δ PS and Δ BCM (B-DAG4); (iv) a path between Δ BCC and both Δ PS and Δ BCM. In this case, Δ BCC is independent from other parameters of community structure (B-DAG5).
(TIF)

Figure S2 **Temporal variability in the rates of change (Δ) in bacterial community diversity (H′ Shannon index, A) and the DGGE banding patterns (B).**
(TIF)

Table S1 **Biotic and abiotic characteristics of the environmental transitions.**
(PDF)

Text S1 **Description of structural equation modeling outputs.**
(PDF)
