# Supplementary figures and images for: Composition Influences the Pathway but not the Outcome of the Metabolic Response of Bacterioplankton to Resource Shifts
Source: PLoS One. 2011 Sep 27;6(9):e25266. doi: 10.1371/journal.pone.0025266 (PMC3181318; doi:10.1371/journal.pone.0025266)

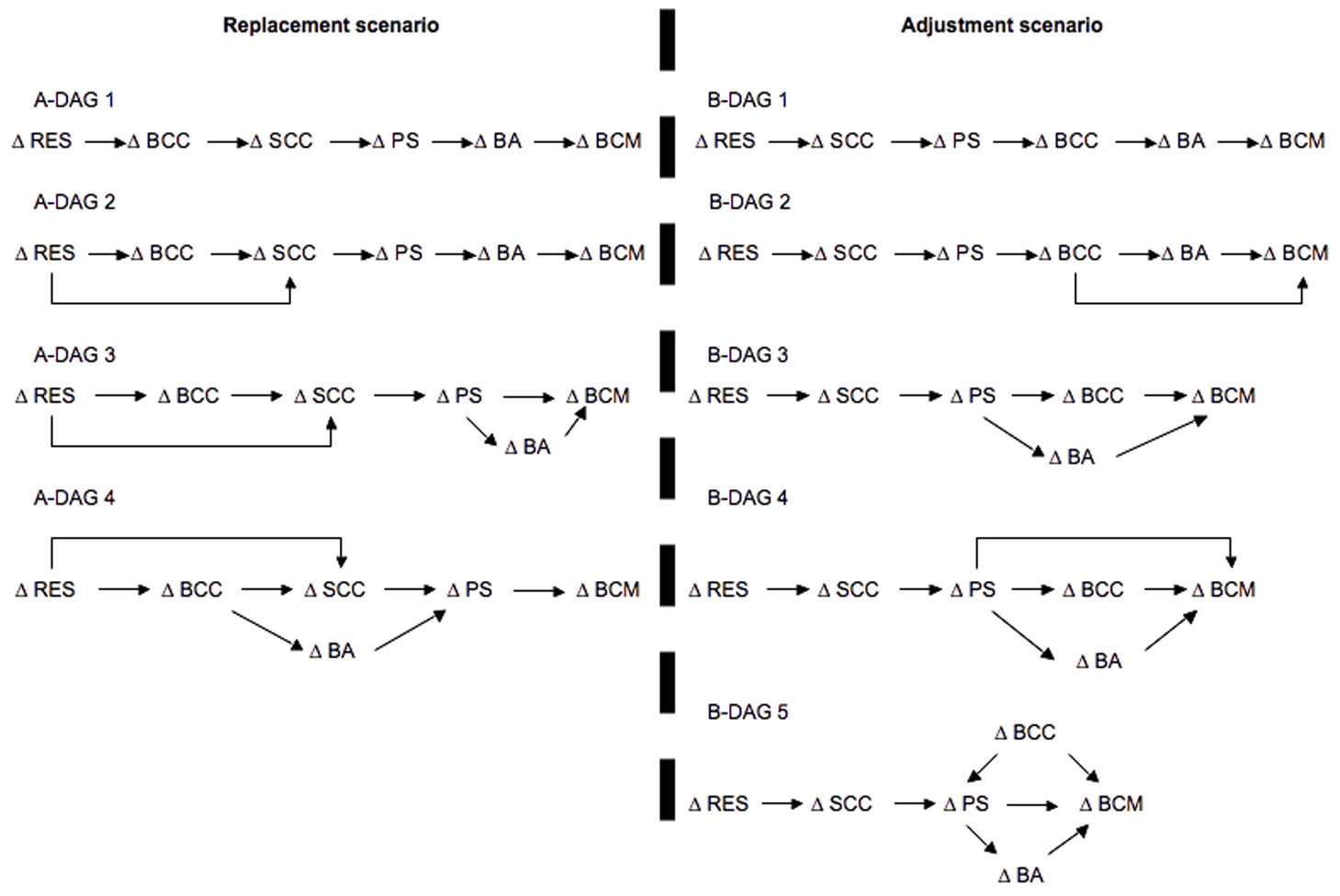

Supplement: Figure S1 — Modeling of bacterial response to changes in resources (Δ RES). Δ BCC, Δ SCC, Δ PS, Δ BA and Δ BCM represent the rates of change in community composition, single-cell characteristics, physiological structure, total abundance and community metabolism respectively. Arrows represent causal links between variables. The same models have been tested with changes in DOC concentration as independent variable. In Scenario A (Replacement scenario), Δ RES induce activation of particular ecotypes that are characterized by single-cell and physiological properties, which directly determine Δ BCM (A-DAG1). Alternative were considered: (i) a direct link between Δ RES and Δ SCC (A-DAG2); (ii) a direct link between Δ PS and Δ BCM (A-DAG3); (iii) a direct link between Δ BCC and both Δ BA and Δ SCC, both of which linked to Δ PS, which in turn determine Δ BCM (A-DAG4). In Scenario B (Adjustment scenario), Δ RES trigger changes in cell activity of the existing phylotypes, which influence the physiological structure (B-DAG1). Alternative were considered: (i) a direct link between Δ BCC and Δ BCM (B-DAG2), (ii) Δ BA mediates the link between Δ PS and Δ BCM independently from Δ BCC (B-DAG3), (iii) a direct link between Δ PS and Δ BCM (B-DAG4); (iv) a path between Δ BCC and both Δ PS and Δ BCM. In this case, Δ BCC is independent from other parameters of community structure (B-DAG5). (TIF) [file pone.0025266.s001.tif]

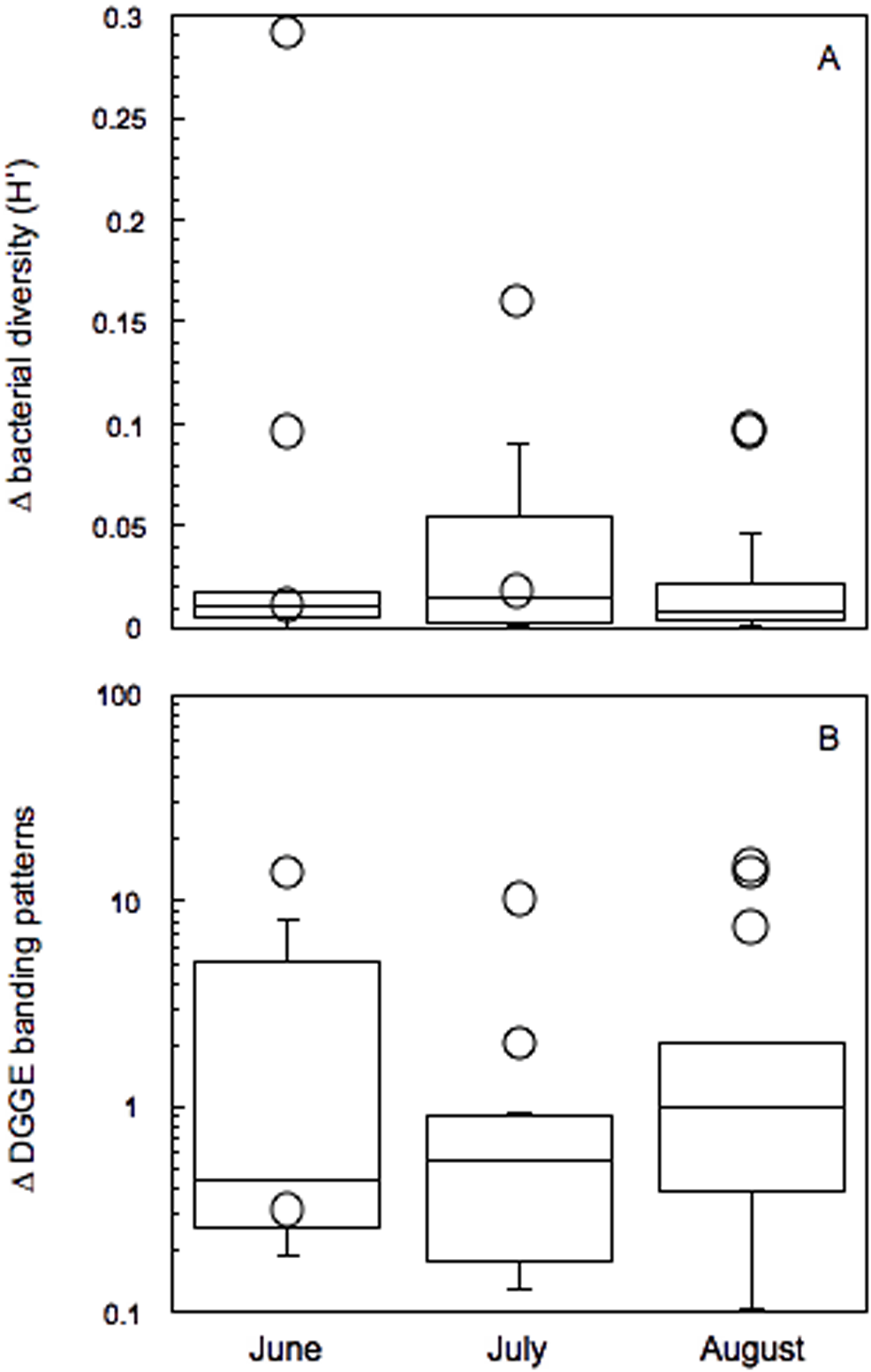

Supplement: Figure S2 — Temporal variability in the rates of change (Δ) in bacterial community diversity (H′ Shannon index, A) and the DGGE banding patterns (B). (TIF) [file pone.0025266.s002.tif]
