# Supplementary material for: Composition Influences the Pathway but not the Outcome of the Metabolic Response of Bacterioplankton to Resource Shifts
Source: PLoS One. 2011 Sep 27;6(9):e25266. doi: 10.1371/journal.pone.0025266 (PMC3181318; doi:10.1371/journal.pone.0025266)
Supplement: Table S1 — Biotic and abiotic characteristics of the environmental transitions. (PDF) [file pone.0025266.s003.pdf]

Table S1. Biotic and abiotic characteristics of the environmental transitions

ET	TT	L	DOC		TP		BP	
			inlet	*output*	*inlet*	*output*	*inlet*	*output*
**1**	0.1	40.6	3.3 (2.6-3.8)	3.7 (3.2-4.3)	4.4 (4.1-4.6)	8.6 (5.7-10.7)	0.29 (0.003-0.7)	0.44 (0.35-0.52)
**2**	19	533.9	3.7 (3.2-4.3)	3.5 (2.2-2.8)	8.6 (5.7-10.7)	1 (0.4-1.3)	0.44 (0.35-0.52)	0.06 (0.03-0.08)
**3**	20	565.1	2.1 (2.1-2.2)	2.8 (2.6-3)	2 (0.9-2.9)	4.7 (2.6-6.3)	0.04 (0.03-0.05)	0.92 (0.7-1.06)
**4**	5.4	153.9	5.7 (4.4-7.9)	5.1 (4.9-5.7)	9.4 (7.1-12)	7.1 (6-8.8)	2.4 (1.76-3.04)	1.07 (0.57-2)
**5**	0.8	863.5	5.1 (4.9-5.7)	5.3 (3.9-7.3)	7.1 (6-8.8)	8.9 (7.1-11.5)	1.07 (0.57-2)	0.77 (0.05-1.52)
**6**	1	2366.8	4.6 (4.5-4.6)	5 (4.5-5.8)	7.3 (4.1-9)	13.1 (7.5-18.3)	0.24 (0.1-0.43)	2.43 (0.36-4.17)
**7**	12	335.3	5 (4.5-5.8)	6.2 (6.1-6.3)	13.1 (7.5-18.3)	10 (7.6-13.1)	2.43 (0.36-4.17)	0.65 (0.15-0.93)
**8**	56	1597.9	4.6 (4.4-4.7)	5.4 (5.1-5.6)	7.6 (4.2-9.7)	7.8 (3.8-13.7)	0.25 (0.15-0.4)	0.69 (0.14-1.49)
**9**	36	1722.5	5.4 (5.1-5.6)	6.4 (5.7-7.2)	7.8 (3.8-13.7)	8.4 (4.4-13.5)	0.69 (0.14-1.49)	1.2 (0.49-1.89)
**10**	22	627.4	6 (4.8-7.1)	6 (6-6.1)	9 (6.6-12.9)	5.3 (4.3-7.4)	2.06 (1.37-2.53)	0.34 (0.23-0.55)
**11**	36	938.4	10 (9-12)	6.1 (5.7-6.4)	21 (20-22)	6 (4.6-8.1)	5.03 (3.12-7.89)	0.25 (0.15-0.38)
**12**	0.1	94.6	5.9 (5.7-6.1)	6.1 (5.7-6.4)	16 (4.7-32.3)	10.6 (4.1-19.2)	1.57 (0.28-3.97)	1.17 (0.38-2.65)
**13**	6.3	175	6.1 (5.7-6.4)	6.7 †	10.6 (4.1-19.2)	10.7 (8.3-12.4)	1.17 (0.38-2.65)	1.62 (0.14-2.98)

ET represents the identity of the environmental transition considered; TT and L refer to the water transit time (hours) and length (meters) of the transitions respectively. DOC and TP refer to concentrations of dissolved organic carbon (mg L^{-1}) and total phosphorus (µg L^{-1}) respectively. BP corresponds to bacterial production rates assessed as uptake of ^{3}H-leucine (µgC L^{-1} hr^{-1}). Values represent mean (minimal-maximal) estimations calculated from 3 replicates excepting ET 13† due to missing values in DOC concentrations.
