# Supplementary material for: Composition Influences the Pathway but not the Outcome of the Metabolic Response of Bacterioplankton to Resource Shifts
Source: PLoS One. 2011 Sep 27;6(9):e25266. doi: 10.1371/journal.pone.0025266 (PMC3181318; doi:10.1371/journal.pone.0025266)
Supplement: Text S1 — Description of structural equation modeling outputs. (PDF) [file pone.0025266.s004.pdf]

Text S1: Description of structural equation modeling outputs.

Structural equation modeling (SEM) is an extension of multiple linear regression models and allows to identify and interpret linear relationships among multiple components (Shipley 2002). Output of SEM analysis is presented in the form of a path diagram (Directed Acyclic Graphs, DAG) that shows the sequence of different components. The structure of DAG presented in this study, are based on both expected connections among the different components, and also from the output of exploratory analyses that suggest most probable correlations among variables using the EPA2 program from Causal Toolbox Package (Shipley 2002). We tested all nine alternative models (Figure S1) for the complete data set using multigroup analysis, which fixes the model structure but allows the free parameters to vary among different groups of data; in this case the groups corresponded to either the three sampling periods allowing us to assess whether the same structure fit the data for the different periods, or high or low level of intensity of environmental gradients. In cases when multigroup analyses yielded no significant structures, we further tested the nine alternative models for each group separately.

In SEM ouputs presented in Figure 5, the r^2 shown above each component represents the proportion of its variability that is explained by the ensemble of preceding components located along the path; the numbers above the arrows refer to the strength of link between two components (i.e. path coefficient). In this study, we have used the standardized path coefficients, i.e. values are standard deviation change in one component given a standard deviation change in the preceding connected component so that these can be compared between each other. Significance of a model is assessed using a χ^2 test that compares the fit between the observed and predicted covariance matrices of the variables (Shipley 2002). The p value indicates the probability of having observed the minimum residual difference between the observed and expected covariances. SEM tests the null hypothesis that the observed and predicted covariances

are identical except for random sampling variation. Thus, when p is smaller than the significance threshold of 0.05, the model should be rejected as an explanation of the observed data (Shipley 2002).

Drawbacks of SEM analyses are on the one hand that these are asymptotic, such that a low number of observations result in a high probability of rejecting a significant structure. To account for this, we used a Monte-Carlo test that corrects the Chi-square likelihood distribution for low number of observations. On the other, the non-normality of variables can also influence the statistics of the models. To correct for that, we used a Sattora-Bentler correction that generates "robust" Chi-square estimates (χ^2). SEM analyses were conducted using EQS 6.1 software (mvsoft).
